# Field cabbage detection and positioning system based on improved YOLOv8n

**DOI:** 10.1186/s13007-024-01226-y

**Published:** 2024-06-20

**Authors:** Ping Jiang, Aolin Qi, Jiao Zhong, Yahui Luo, Wenwu Hu, Yixin Shi, Tianyu Liu

**Affiliations:** College of Mechanical and Electrical Engineering, Hunan Agricultural University, Changsha, 410128 China

**Keywords:** Cabbage, Object detection, YOLOv8n, Swin transformer, Large kernel convolutions

## Abstract

**Background:**

Pesticide efficacy directly affects crop yield and quality, making targeted spraying a more environmentally friendly and effective method of pesticide application. Common targeted cabbage spraying methods often involve object detection networks. However, complex natural and lighting conditions pose challenges in the accurate detection and positioning of cabbage.

**Results:**

In this study, a cabbage detection algorithm based on the YOLOv8n neural network (YOLOv8-cabbage) combined with a positioning system constructed using a Realsense depth camera is proposed. Initially, four of the currently available high-performance object detection models were compared, and YOLOv8n was selected as the transfer learning model for field cabbage detection. Data augmentation and expansion methods were applied to extensively train the model, a large kernel convolution method was proposed to improve the bottleneck section, the Swin transformer module was combined with the convolutional neural network (CNN) to expand the perceptual field of feature extraction and improve edge detection effectiveness, and a nonlocal attention mechanism was added to enhance feature extraction. Ablation experiments were conducted on the same dataset under the same experimental conditions, and the improved model increased the mean average precision (mAP) from 88.8% to 93.9%. Subsequently, depth maps and colour maps were aligned pixelwise to obtain the three-dimensional coordinates of the cabbages via coordinate system conversion. The positioning error of the three-dimensional coordinate cabbage identification and positioning system was (11.2 mm, 10.225 mm, 25.3 mm), which meets the usage requirements.

**Conclusions:**

We have achieved accurate cabbage positioning. The object detection system proposed here can detect cabbage in real time in complex field environments, providing technical support for targeted spraying applications and positioning.

## Introduction

Crop diseases and pests pose significant threats to agricultural production, affecting crop yields and quality and leading to shortages in the food supply. Farmers primarily use agricultural chemicals to control plant diseases, pests, and weeds despite their negative impact on the environment and human health. Reducing the adverse effects of agricultural chemicals is a major societal challenge worldwide [1]. Compared to traditional continuous uniform spraying methods, targeted spraying is a more environmentally friendly alternative method that can reduce pesticide pollution and costs and improve spraying effectiveness. To spray cabbage in a targeted manner, a cabbage positioning system needs to be built for the spraying equipment. Detection is a prerequisite for positioning technology, and common target detection technologies in agricultural fields include machine vision, ultrasonic sensors [2], and 3D laser radar [3, 4]. However, ultrasonic sensors and 3D laser radar struggle to accurately distinguish crops from weeds in large fields. Machine vision technology, with the benefits of large information acquisition, high accuracy, and intelligence, has potential advantages in cabbage recognition [5].

This article describes the use of deep learning for cabbage detection because it is difficult to achieve good accuracy when manually extracting features from photos captured in highly complex agricultural environments. A convolutional neural network (CNN) can discover more abstract and hidden features in images, thus improving performance, reducing the manual workload, and realizing target positioning.

Since the introduction of the AlexNet algorithm [6] in 2012, deep learning has gradually become mainstream, with various types of deep learning algorithms constantly emerging. Currently, deep learning-based object detection algorithms can be divided into two main types: one-stage and two-stage algorithms. Notable one-stage algorithms include the YOLO series [7–11] and SSD [12], while the mainstream two-stage algorithms include R-CNN, Fast R-CNN, and Faster R-CNN [13–15]. One-stage algorithms have a clear advantage in terms of detection speed, while two-stage algorithms often achieve better detection accuracy. Due to the complexity of agricultural environments, directly applying the abovementioned object detection algorithms often fails to yield satisfactory results; thus, extensive research has been conducted to improve outcomes.

The inference process of deep learning models requires significant computational resources, whereas mobile devices typically have lower computational capabilities. Currently, one of the main challenges with the use of mobile devices is balancing speed and accuracy. Ong [16] and others formerly performed weed detection among commercial Chinese cabbage crops using images acquired by unmanned aerial vehicles. The acquired images were preprocessed and subsequently segmented into crop, soil, and weed classes using the simple linear iterative clustering superpixel algorithm. The segmented images were then used to construct the CNN-based classifier, and random forest (RF) was applied to compare its performance with that of the CNN. To effectively and accurately identify field vegetables and weeds, Ma [17] and others proposed a semantic segmentation model called MSECA-Unet based on an improved U-Net architecture. This model significantly reduces the number and size of the parameters by introducing multiscale inputs and a nonlocal attention mechanism, achieving rapid identification of cabbage crops and weeds. Ye [18] and others compared the performance of two advanced methods, DL and OBIA, in individual cabbage plant detection tasks. The results show that the Mask R-CNN deep learning model outperforms the object-based image analysis multilevel distance transform watershed segmentation method in crop extraction and counting, with an overall mean F1 score and accuracy that are 2.70 and 4.15% greater, respectively. Sun [19] and others proposed a cabbage transplantation state recognition model based on YOLOv5-GFD. Compared to the original model, the mean average precision (mAP) increased by 3.5%, the recall increased by 1.7%, and the detection speed increased substantially by 52 FPS.

These studies demonstrate the various approaches that have been applied to address the challenge of balancing speed and accuracy in deep learning models for cabbage detection on mobile devices. From the above literature, it can be seen that there has been relatively little research on the comprehensive detection of cabbage growth processes, and there is considerable room for improvements in positioning accuracy. Considering the acceptable range in precision of target spray positioning for mechanical transplanting of cabbage in actual spraying processes and the characteristics of complex natural environments and severe cabbage occlusion during mature stage, in this paper, a field cabbage position recognition algorithm, YOLOv8-cabbage, based on improved YOLOv8n, is proposed. This algorithm incorporates three strategies for improvement large kernel convolution, a Swin transformer module, and an attention mechanism to enhance the detection accuracy. Additionally, this algorithm is combined with a depth camera to obtain the real-time three-dimensional coordinates of cabbages. The results indicate that the error of this algorithm is within an acceptable range.

## Materials and methods

### Data acquisition and preprocessing

#### Cabbage dataset

**Fig. 1:**
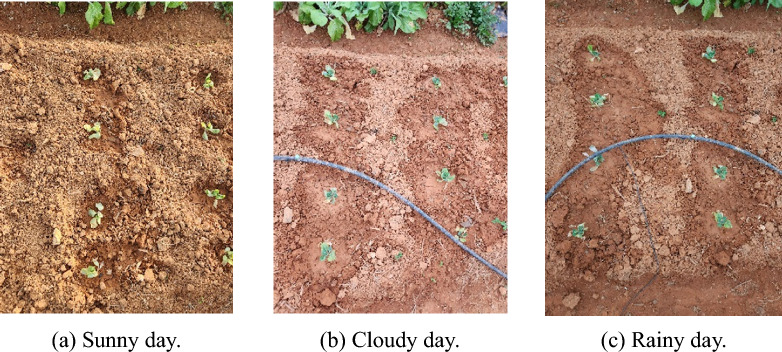
Images acquired under different environmental conditions

**Fig. 2:**
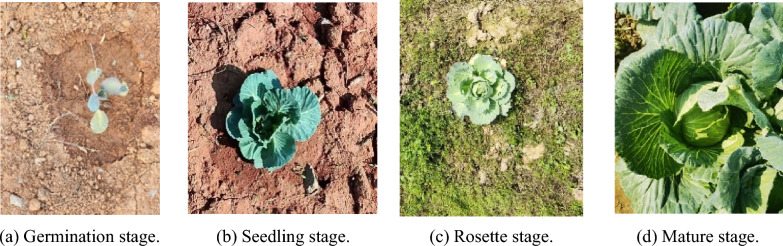
Cabbage in different growth stages

The height of the camera during the collection of the dataset was set to around 70 cm. This height fully considers the size of the existing spraying equipment and the installation position of the camera. The images and videos were collected vertically downwards, with a resolution of 3000 pixels × 4000 pixels. The dataset for this experiment has three main sources: The first part comes from the open-air vegetable experiment base of Hunan Agricultural University. It mainly collects images of cabbage from germination to seedling stage. From November 12 to December 30, 2023, images were collected every 15 days starting from the 7th day after planting, for a total of three times. To ensure the diversity of the sample environment, the lighting conditions included sunny, cloudy, and rainy days. A total of 1087 images were collected, and the images taken under different weather conditions are shown in Fig. 1. Each image contains multiple cabbage targets and various weeds, reflecting the real scene. The second part of the data includes 400 images taken in the field of Wangcheng District, Changsha City by Jinxiu Ecological Agriculture Co., Ltd. These images cover all growth stages of cabbage, filling the gap of rosette stage and mature stage in the first part. Images of different growth stages are shown in Fig. 2. The third part consists of 159 images from the Internet, which are used to improve the robustness of the model. This part of the data is composed of images taken under different environmental conditions with different pixel sizes.

#### Data preprocessing

**Fig. 3:**
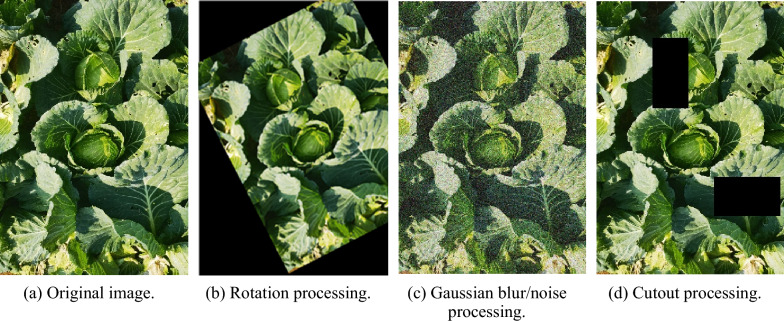
Illustrations of the dataset augmentation

To enhance the richness of the dataset, this study employed computer vision techniques for data augmentation on the original images (Fig. 3a). The techniques used in this study include image rotation (Fig. 3b), Gaussian blur/noise (Fig. 3c), and cutout (Fig. 3d) processing [20]. The cutout technique involves randomly deleting multiple rectangular regions. After deleting important regions, the model relies on other information for classification, which results in better model generalizability.

Through these three techniques, the dataset was expanded threefold, and manual labelling was performed using LabelImg software in YOLO file format. Considering the significant morphological changes in the cabbage during the mature stage and the requirements for pesticide application, the images taken after the cabbage had entered the mature stage were labelled as mature cabbage, while those not yet in the mature stage were labelled as immature cabbage to improve the accuracy of the detection model. The dataset was further divided into a training set (80%), a validation set (10%), and a testing set (10%). The training set was used for model fitting, the validation set was used to adjust the hyperparameters used during training and for preliminary evaluation of the model’s capabilities, and the testing set was used to evaluate the generalizability of the final model.

### Cabbage detection CNN

#### YOLOv8

**Fig. 4:**
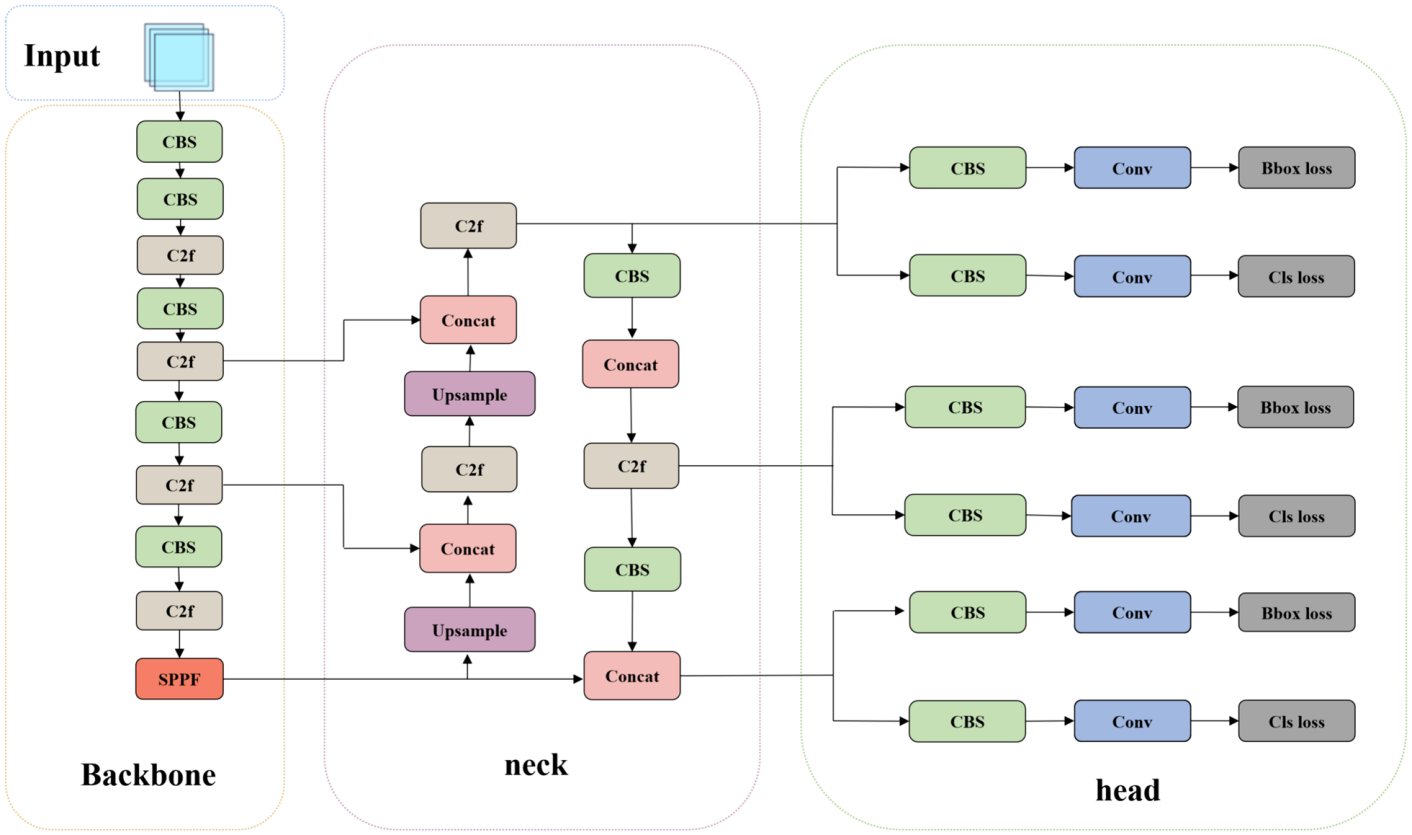
Overall architecture of the YOLOv8n

This study conducted a comparative analysis and Preliminary experiment on four mainstream object detection models, Faster R-CNN, YOLOv5s, SSD, and YOLOv8n, to determine the most suitable transfer learning model for cabbage detection. Faster R-CNN differs from other models in that it has an additional step at the beginning to generate region proposals, which is typically time-consuming, followed by feature extraction and classification. SSD works by generating many boxes of different shapes and sizes for each pixel, and then selecting the appropriate boxes as the detection results after feature extraction and classification. YOLO, on the other hand, first divides the image into grids and then generates a small number of boxes on each grid, further reducing the computational cost. YOLOv8 replaces the C3 structure in YOLOv5 with the C2f structure, which has richer gradient flow, and adjusts the number of channels for different scale models. This enables even very small models like YOLOv8n to achieve good detection performance, especially for images captured by mobile devices with fewer image pixels. Compared with other lightweight detection models, YOLOv8n demonstrated the fastest detection speed and the best detection performance on the dataset. Therefore, YOLOv8n was selected and optimized to construct a detection model capable of quickly and accurately identifying cabbage targets in complex field environments. YOLOv8 is an object detection model that was released by Ultralytics on January 10, 2023. The detailed architecture of YOLOv8n is shown in Fig. 4.

The architecture of YOLOv8 consists of the following components:

- Backbone: Responsible for extracting feature information from the image and providing input to the subsequent networks. CBS is a standard convolutional module that extracts features. The C2f module extracts and fuses features using concat fusion, which is a type of residual connection, with features from different bottleneck layers. SPPF fuses features that have not been maxpooling or have been maxpooling at most three times. After continuous fusion and convolution through these modules, the backbone can provide an image with rich feature information.
- Neck: This layer is located between the backbone and the head, aiming to better utilize the features extracted by the backbone network for feature fusion. The neck network employs numerous concat and C2f modules to fuse low-level feature maps with high-level feature maps that have undergone extensive convolutions.
- Head: This layer utilizes previously extracted features for object detection. The Bbox loss module is responsible for bounding box regression, while the Cls loss module is responsible for category classification.

#### Large kernel convolutions

To address the issue of the model's poor adaptability to detecting cabbages in different growth stages and under different conditions, we propose using larger convolution kernels to attempt to solve this problem. Typically, dilated convolutions come with high computational costs because the size of the convolutional kernel is directly proportional to the number of parameters and floating-point operations. However, this drawback can be mitigated by using deep convolutions [21]. The computational formula for standard convolutions is as follows.

1 $$FLOPs=\left(2\times C\times {K}^{2}-1\right)\times H\times W\times {C}^{^{\prime}}$$

Additionally, the calculation formula for the computational complexity of depthwise convolution is:

2 $$FLOPs=C\times {K}^{2}\times H\times W$$

where $FLOPs$ represents the number of floating-point operations, $W$ represents the number of pixel columns in the image, $H$ represents the number of pixel rows in the image, $C$ represents the number of input channels in the image (or feature map), ${C}^{^{\prime}}$ is the number of output feature maps, and $K$ indicates the size of the convolutional kernel.

**Fig. 5:**
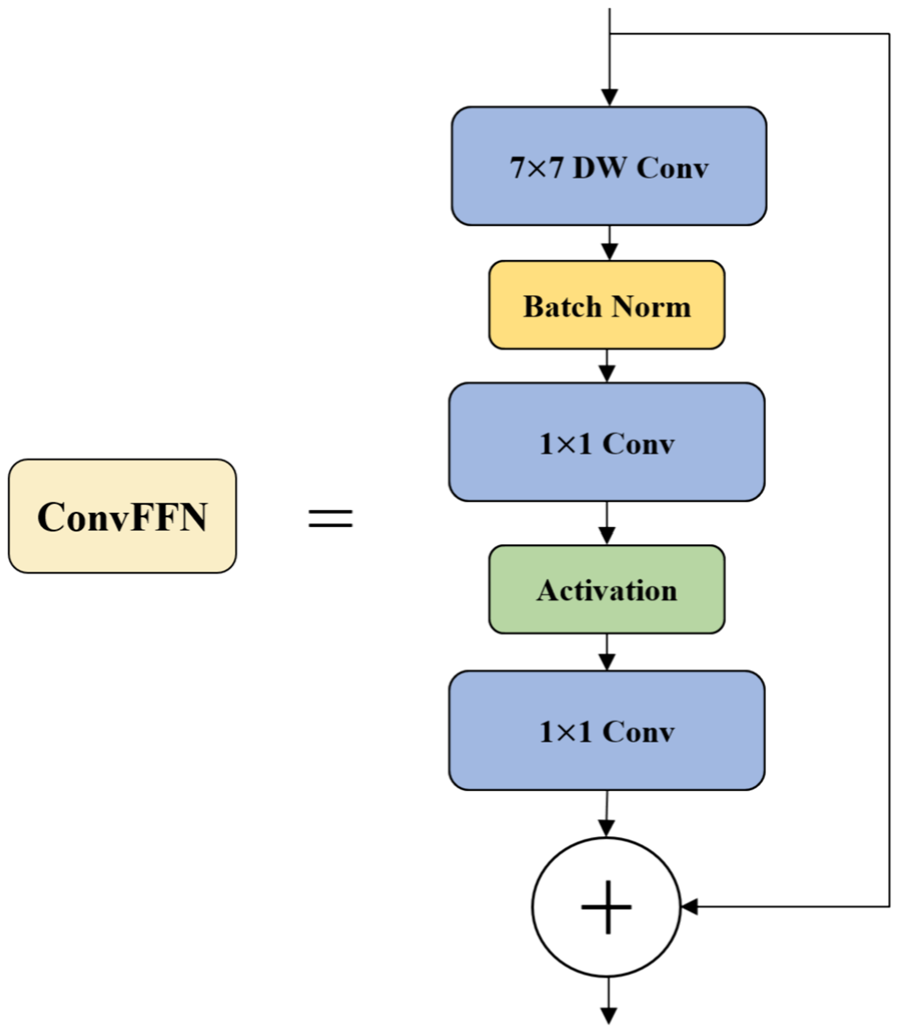
Illustration of the ConvFFN

**Fig. 6:**
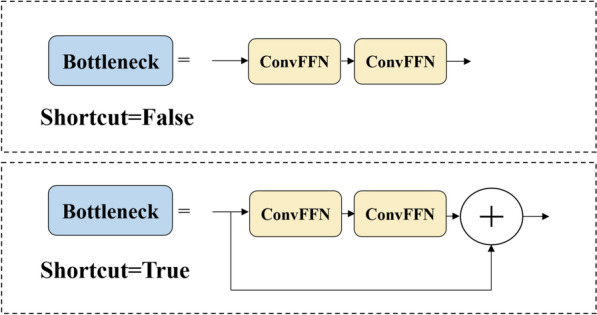
Improved bottleneck module

During deep convolution, each convolutional kernel acts on each channel of the input image or feature map. Using deep convolutions results in less growth among floating-point operations and fewer parameter counts. Dilated convolutions allow a larger receptive field, which can have an impact on downstream tasks [22]. Additionally, the design of dilated convolutions introduces more shape biases into the network, helping to improve the model's generalizability and reduce the risk of overfitting [23]. Therefore, a set of dilated convolutional neural network (ConvFFN) modules was used, as shown in Fig. 5, to replace the convolution modules in the bottleneck module. The improved bottleneck module is shown in Fig. 6. By implementing these modifications, we anticipate an improvement in detection accuracy while incurring a minimal decrease in detection speed.

#### Swin transformer

Due to their inherent characteristics, CNNs exhibit relatively weak responses at the edges of images. This is because the pixels located at the edges of images contribute less to gradient updates due to fewer convolution operations, resulting in poor cabbage detection performance in the edge regions of images. Adding a Swin transformer detection layer to the original network allows the improved model to overcome the limitations of CNN convolution operations, enhancing edge detection performance and recognizing abstract information from low-level features, thereby strengthening the semantic information about the cabbage during the seedling stage [24–26].

**Fig. 7:**
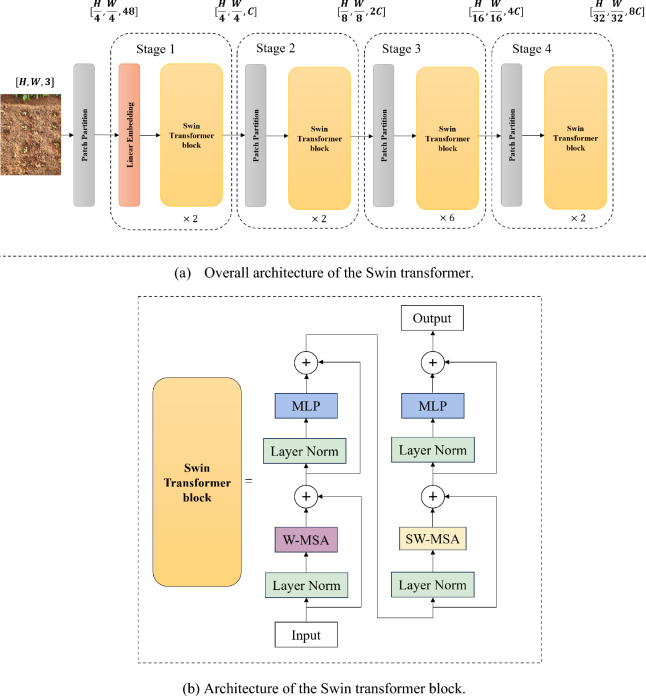
Illustration of the Swin transformer

Figure 7 provides an overview of the Swin transformer architecture. First, it utilizes the patch partition module to partition the input RGB image into dimensions of $H\times W\times 3$, where each 4 $\times$ 4 adjacent pixels form a patch. Assuming that the input is an RGB three-channel image, each patch contains 4 $\times$ 4 = 16 pixels, and subsequently, each pixel has three values (R, G, B); i.e., a feature dimension of 16 $\times$ 3 = 48. After patch partitioning, the shape of the image changes from [H, W, 3] to [H/4, W/4, 48].

Next, the linear embedding layer performs linear transformations on the channel data of each pixel. The feature maps are then fed into four self-attention transformer blocks, generating a hierarchical representation. The Swin transformer does not require pooling or other downsampling methods to reduce the size of the feature maps, which thereby prevents information loss.

#### Nonlocal attention

Significant breakthroughs have been made in recent years in the attention mechanisms of various fields, such as image processing, natural language processing, and computer vision, which have been suggested to be beneficial for improving model performance. The marked effectiveness of channel or spatial attention mechanisms in generating more discernible feature representations is evident in various computer vision tasks. However, modelling cross-channel relationships with channel dimensionality reduction may have unwanted effects on extracting deep visual representations.

**Fig. 8:**
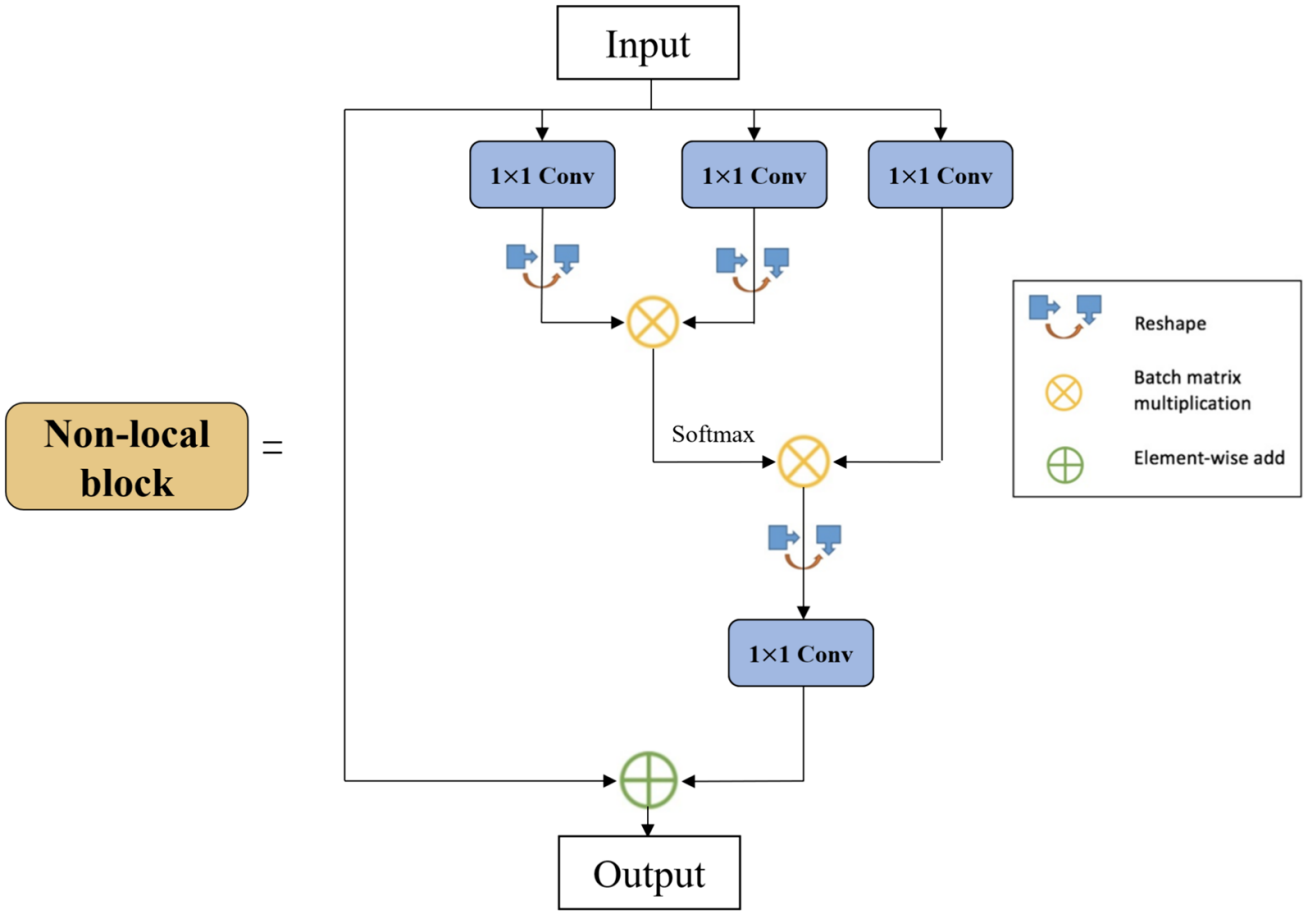
Illustration of the nonlocal block

Fundamentally, the aim of spatial domain attention methods is to transform spatial information from the original image into another space while preserving key information. This approach avoids the potential unwanted effects that may arise from reducing dimensions using channel attention mechanisms [27–29]. The nonlocal attention module is shown in Fig. 8.

#### Overall structure of the cabbage detection model

**Fig. 9:**
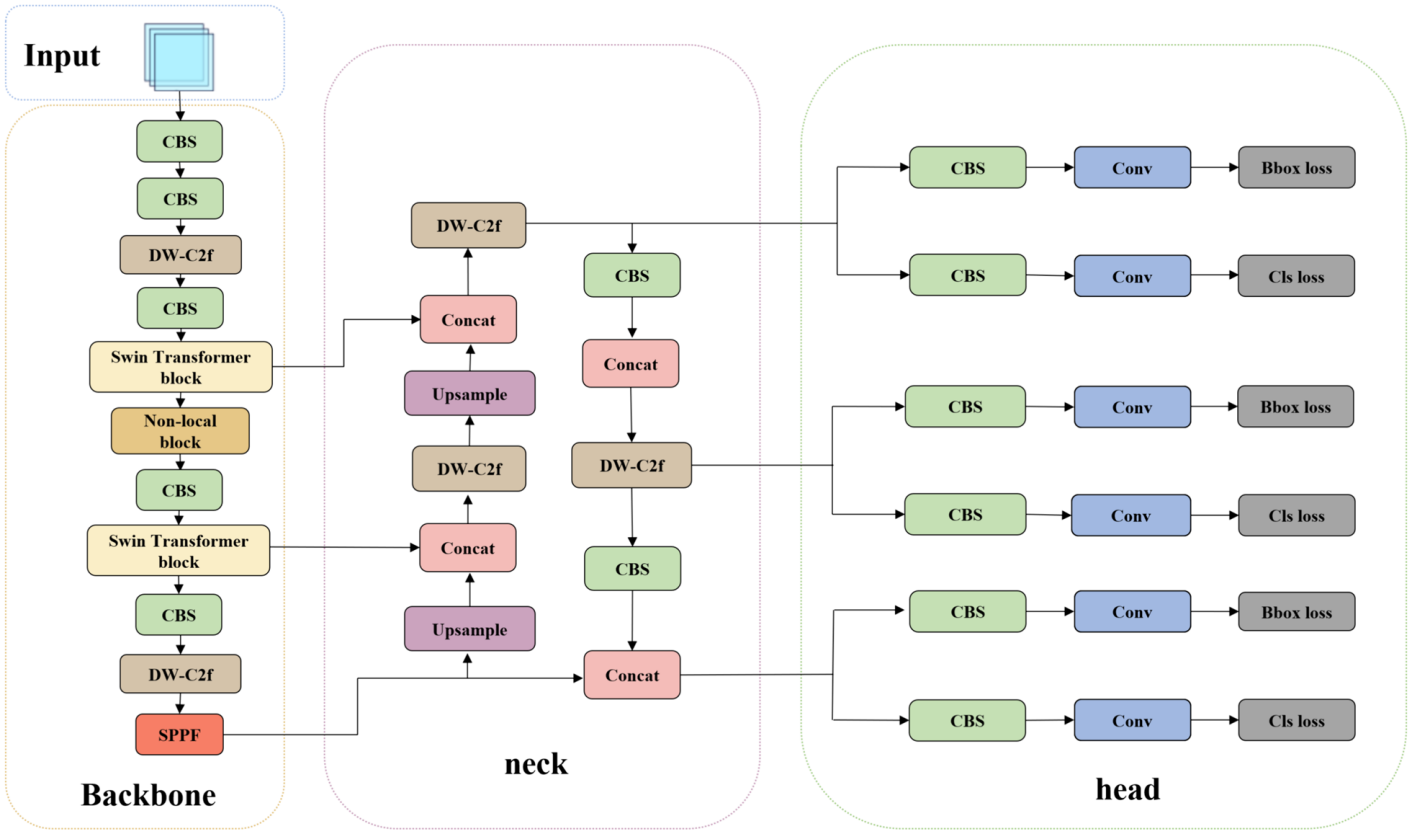
Overall architecture of the YOLOv8-cabbage

Our proposed fusion network, YOLOv8-cabbage, is shown in Fig. 9, which has been improved to address challenges that may arise during field detection.

First, the ConvFFN structure replaces the convolution module in the bottleneck module. By employing large kernel convolution modules in C2f, we increase the receptive field to enhance the overall robustness of the model and reduce the risk of overfitting. The C2f module modified by this method is here renamed DW-C2f. Placing the attention mechanism in the middle layer of the network allows for a better combination of low-level and high-level features. Therefore, we inserted the nonlocal attention mechanism module after the original second C2f module. To address the challenges in small target detection and edge detection, we replaced the original second and third C2f layers of the backbone network with the Swin transformer module.

## Results and discussion

### Cabbage detection experiment

#### Experimental conditions

The model training platform is a desktop workstation with the following configuration: 16 GB of memory, equipped with an AMD Ryzen 7 5800 × CPU, and an NVIDIA RTX 4060 GPU. The operating system is Windows 11 (64-bit), the programming language is Python version 3.10.13, the CUDA version is 11.8, the compiled IDE is PyCharm, and the deep learning framework is PyTorch 2.0.1. The learning rate is set to 0.01, the optimizer is Adam, the batch size is set to 16, and the experiment is configured with 300 iterations (epoch).

#### Evaluation criteria

To comprehensively assess the model performance, the precision (P), recall (R), mAP, and average image processing time are adopted as the evaluation parameters. mAP, is the average value of the average precision (AP) and a primary evaluation metric for object detection algorithms. Object detection models are often described in terms of speed and accuracy (mAP). A higher mAP indicates better detection performance on the given dataset. This paper uses mAP@0.5, meaning that the target is a confidence level that exceed 0.5.

#### Mainstream model test results

On the cabbage dataset, we conducted experiments with Faster R-CNN, SSD, YOLOv8n, and YOLOv5s, and these results are listed in Table 1. We observed that Faster R-CNN has an advantage in terms of detection accuracy, but its detection speed is slower, making real-time detection challenging for further research and applications. The average processing time for a single-frame image in YOLOv8n is 20.1 ms, which is only 14.8% of the time required by Faster R-CNN, with a mAP of 88.8%, an improvement of 2.7% compared to YOLOv5s. Overall, in terms of balancing cabbage recognition accuracy and image processing speed, YOLOv8n has certain advantages over the other networks tested.

**Table 1:** Mainstream model performance test results

Model	mAP@0.5 (%)	Precision (%)	Recall (%)	Average image processing time (ms)
YOLOv5s	86.1	86.7	80.0	25.7
YOLOv8n	88.8	91.9	78.1	20.1
SSD	81.9	79.9	72.2	29.4
Faster R-CNN	91.4	89.2	82.0	135.6

#### Ablation experiments

A series of ablation experiments were conducted to validate the performance of the improved algorithm, and tests were carried out on a self-built dataset. The experiments included data augmentation, the addition of nonlocal attention modules, ConvFFN large kernel convolution modules, and Swin transformer modules. These results are shown in Table 2. All networks used models pretrained on the COCO dataset [30]. The training and validation datasets were kept consistent across all experiments to control variables and ensure the validity of the results.

**Table 2:** Results of the ablation experiments

Group	Image preprocessing	ConvFFN	Nonlocal	Swin transformer	Precision (%)	Recall (%)	mAP@0.5 (%)	Detection time (ms)
1	×	×	×	×	85.4	75.8	86.0	20.1
2	√	×	×	×	91.9	78.1	88.8	20.1
3	√	√	×	×	91.3	83.3	91.3	20.7
4	√	√	√	×	92.6	83.0	91.9	23.5
5	√	√	√	√	95.5	85.1	93.9	26.3

* “√” indicates that the current network uses this structure or method; X indicates that the structure or method is not in use on the current network

From Table 2, the average accuracy of the original model increased from 86.0% to 88.8% when trained on an image dataset with data augmentation compared to without data augmentation. When the original model and YOLOv8-cabbage were both trained on an image dataset with data augmentation, the mAP increased from 88.8% to 93.9%, with precision and recall increasing by 3.6% and 7%, respectively. By enhancing the data and introducing an attention module, particularly one that focuses on the edge features of cabbage images, improvements were achieved. The combination of the global attention advantage of the Swin transformer module and the feature extraction advantage of the large convolutional kernel enhances the network's performance in extracting feature information.

**Fig. 10:**
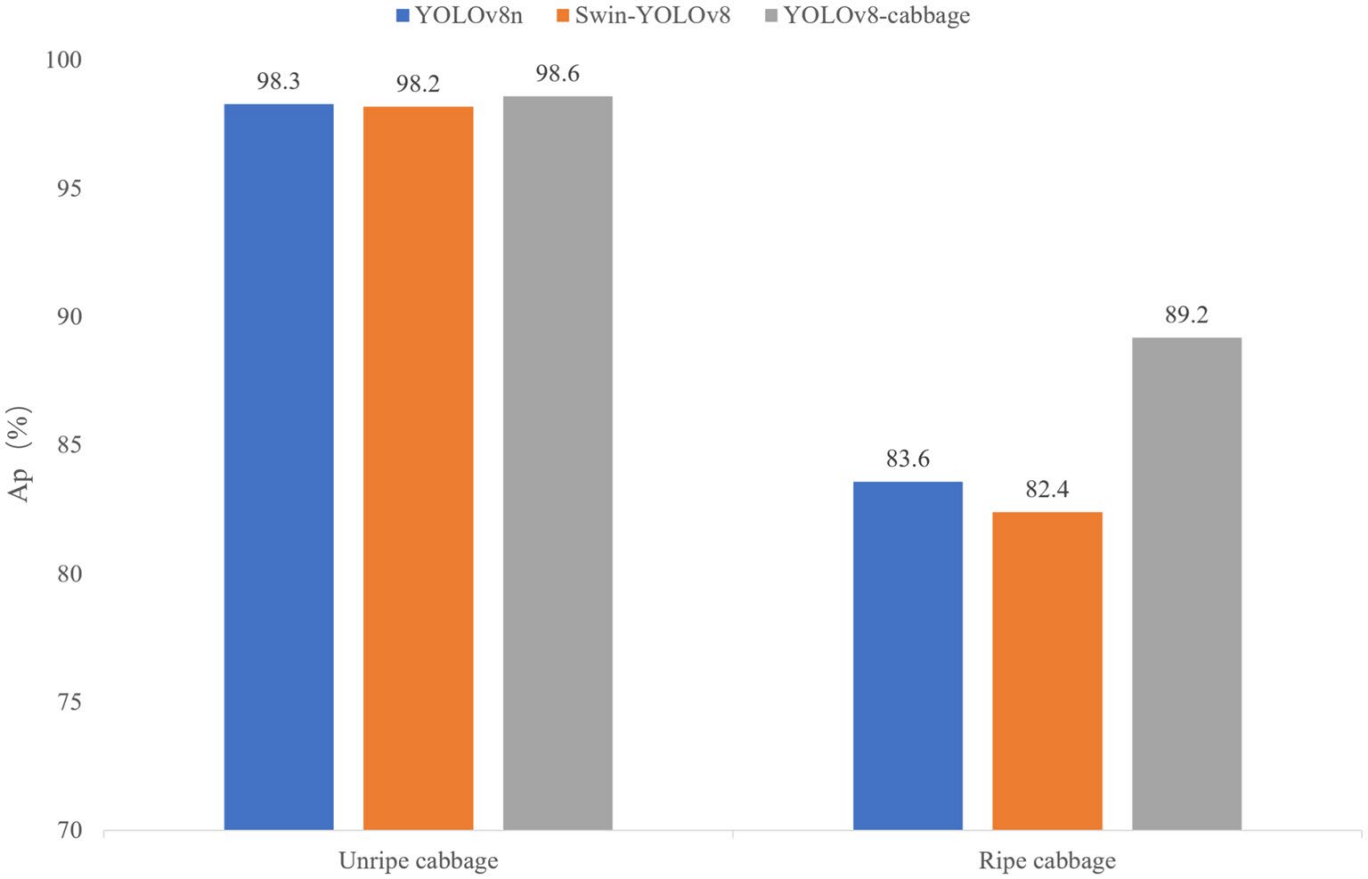
Comparison of three structural models on the cabbage dataset

Given that the improved model ensures a higher AP and a processing speed suitable for practical use, we further aimed to verify that the improved algorithm, which combines the global attention advantage of the Swin transformer module with the feature extraction advantage of the large convolutional kernel, outperforms models that rely solely on either convolutional feature extraction or Swin transformer feature extraction. Furthermore, we compared the YOLOv8-cabbage model with the original model that included only the Swin transformer; these test results are shown in Fig. 10. YOLOv8-cabbage achieves higher detection accuracy than the other two models.

#### Test results

**Fig. 11:**
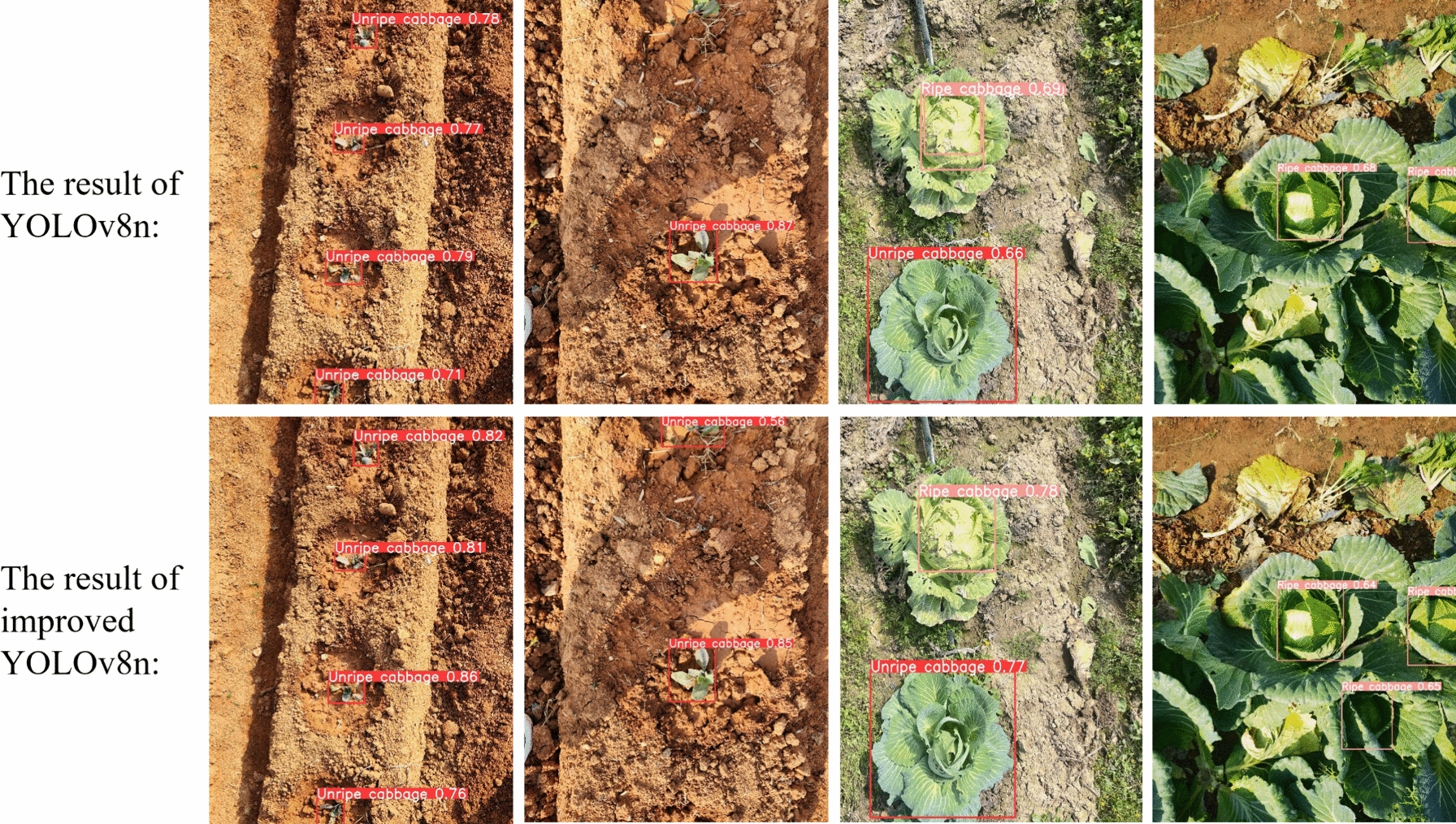
Comparison of detection results before and after YOLOv8n network improvement

Figure 11 shows a comparison of the detection results before and after the YOLOv8n network was improved using the same validation set images. In both sets of detection results, it can be observed that the improved model not only achieves better confidence levels in simple detection tasks but also avoids missing detections at the image edges. There are fewer instances of repeated detection under sufficient lighting conditions, and the occurrence of missed detections is significantly reduced under conditions of insufficient lighting or severe occlusion.

### Cabbage positioning experiment

#### Positioning methods and camera calibration

**Fig. 12:**
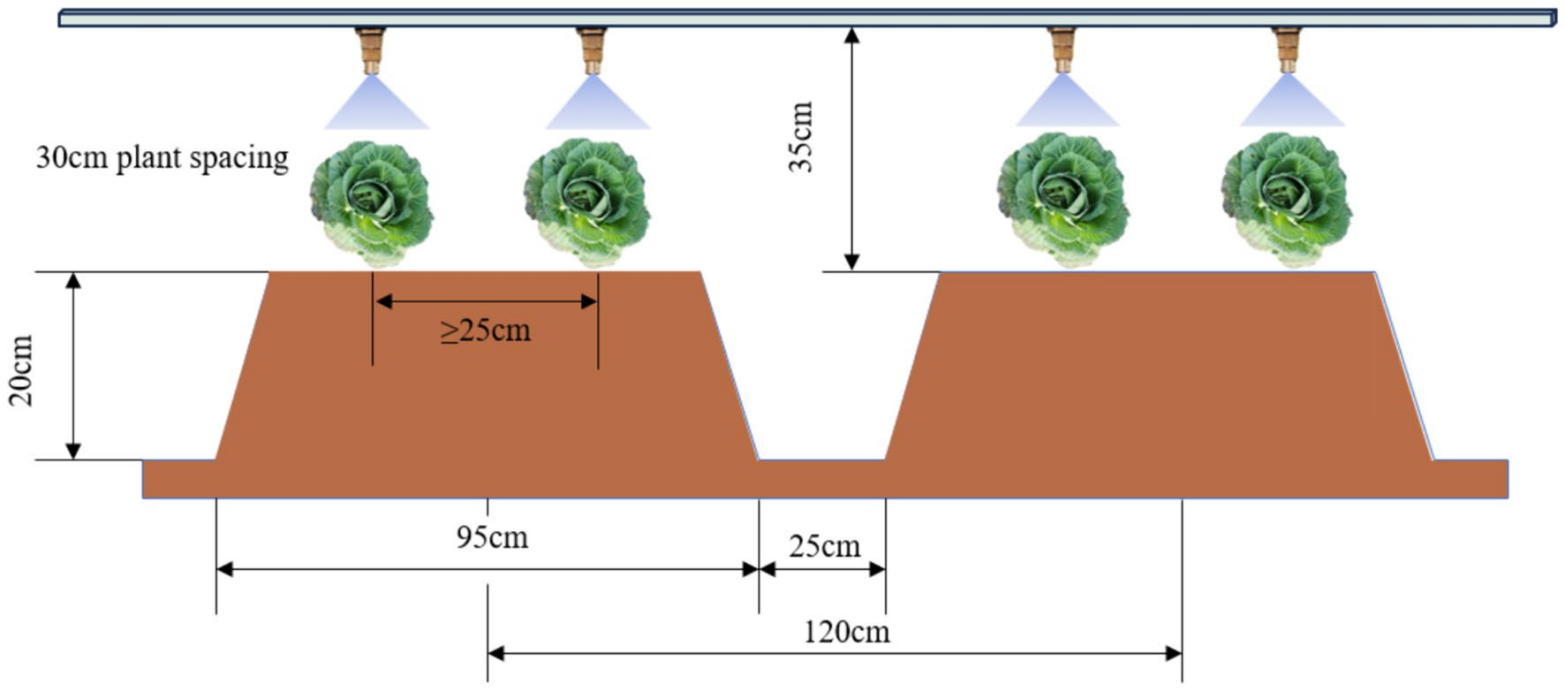
Working diagram of the existing spraying equipment

This article uses the mechanized production of cabbage vegetables proposed at the 2023 China Vegetable Industry Conference as the standard and measures the allowable error range of three-dimensional coordinates based on the growth and planting characteristics of cabbage. As shown in Fig. 12, the existing spraying equipment nozzle sprays chemicals at a 110° angle, with the minimum vertical height of the connecting rod to the nozzle being 35 cm. When operating at this minimum height, with a standard ridge height of 20 cm, the spraying radius is 21.45 cm. Measurements of 20 groups of cabbage plants during the seedling stage revealed an average radius of 9 cm. Therefore, there is an error range of 12.45 cm in the XY plane, and at a distance of 6.3 cm from the ridge, a radius of 9 cm can be sprayed. In summary, the maximum X-axis and Y-axis coordinate errors are 88 mm, and the maximum Z-axis coordinate error is 87 mm.

This article uses Intel’s Realsense D435i series stereo camera for research. The camera consists of left and right infrared cameras for depth measurement and an RGB camera for capturing colour images. After calibrating the camera, improved models are used to obtain the coordinates of the centre point of the colour image. Then, in the depth image aligned with the colour image, the depth value at the corresponding pixel point of the target can be obtained, thus achieving three-dimensional spatial positioning of the cabbage.

**Fig. 13:**
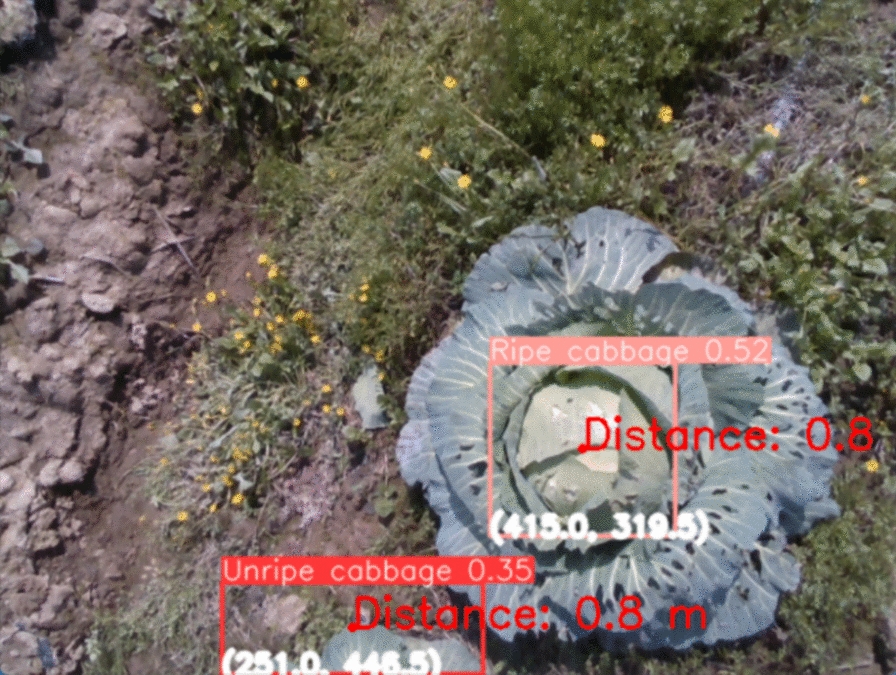
Cabbage anchor point diagram

The spatial coordinates outputted by the midpoint of the cabbage detection box in the pixel coordinate system uv are used as the measurement position for cabbage-targeted spraying, as shown in Fig. 13, with the bottom coordinates of the detection box corresponding to the coordinates of the red dot in the detection box.

#### Camera calibration

The GML Calibration Toolbox and Intel RealSense Viewer were used to view the intrinsic parameters. The colour and depth cameras were calibrated using a dynamic calibration board with fixed dimensions to obtain the camera’s intrinsic and distortion parameters. Using Zhang’s camera calibration method, after obtaining an image of the calibration board, the corresponding image detection algorithm can be used to obtain the pixel coordinates of each corner point. Zhang’s calibration method fixes the world coordinate system on the checkerboard, and the size of each grid on the checkerboard is known. We can calculate the physical coordinates of each corner point in the world coordinate system. The camera intrinsic matrix can be solved by using the pixel coordinates and physical coordinates. The results of the camera calibration are shown in Table 3.

**Table 3:** Internal parameters and distortion parameters of the binocular camera

Argument	Camera calibration result	
	Depth camera	Colour camera
Focal length		
${f}_{\text{x}}$/pixel	636.174	954.261
${f}_{y}$/pixel	636.174	954.261
Principal point		
${C}_{\text{x}}$/pixel	640.706	961.077
${C}_{y}$/pixel	356.945	535.417
Rotation matrix	$$\left[\begin{array}{ccc}-0.999& -0.013& -0.004\\ 0.013& 0.999& -0.001\\ 0.004& 0.001& 0.999\end{array}\right]$$	
Translation vector	$[-0.014,-0.0001,-0.0001]$	
Distortion	$[1.0171, 1.0116,-0.1046,-0.0232]$	

In the pixel coordinate system, the image is not in the ideal position ${p}^{^{\prime}}=[{u}^{^{\prime}},{v}^{^{\prime}}]$, so it is necessary to compensate for nonlinear distortion based on the actual position $p=[u,v]$. Radial distortion occurs during camera production due to uneven thickness. After correcting for radial distortion, the position ${p}_{0}^{^{\prime}}=[{u}_{0}^{^{\prime}},{v}_{0}^{^{\prime}}]$ can be expressed as follows:

3 $$\left[\begin{array}{c}{u}_{0}^{\prime}\\ {v}_{0}^{\prime}\end{array}\right]=\left(1+{k}_{1}{r}^{2}+{k}_{2}{r}^{4}+{k}_{3}{r}^{6}\right)\left[\begin{array}{c}u\\ v\end{array}\right]$$

where $r$ represents the curvature radius, ${k}_{1},{k}_{2},{k}_{3}$ represent the radial distortion coefficient, and ${p}_{1},{p}_{1}$ represent the tangential distortion correction coefficient. Due to the nonparallelism between the imaging plane and the lens plane, tangential distortion occurs. After correcting for tangential distortion, the position ${p}_{1}^{^{\prime}}=[{u}_{1}^{^{\prime}},{v}_{1}^{^{\prime}}]$ can be expressed as follows.

4 $$\left[\begin{array}{c}{u}_{1}^{\prime}\\ {v}_{1}^{\prime}\end{array}\right]=\left[\begin{array}{c}2{p}_{1}uv+{p}_{2}\left({r}^{2}+2{u}^{2}\right)\\ 2{p}_{2}uv+{p}_{1}\left({r}^{2}+2{v}^{2}\right)\end{array}\right]$$

Since both types of distortion occur independently in the lens, they need to be considered simultaneously, as follows.

5 $$\left[\begin{array}{c}{u}^{\prime}\\ {v}^{\prime}\end{array}\right]=\left[\begin{array}{c}{u}_{0}^{\prime}\\ {v}_{0}^{\prime}\end{array}\right]+\left[\begin{array}{c}{u}_{1}^{\prime}\\ {v}_{1}^{\prime}\end{array}\right]$$

#### Coordinate system transformation

**Fig. 14:**
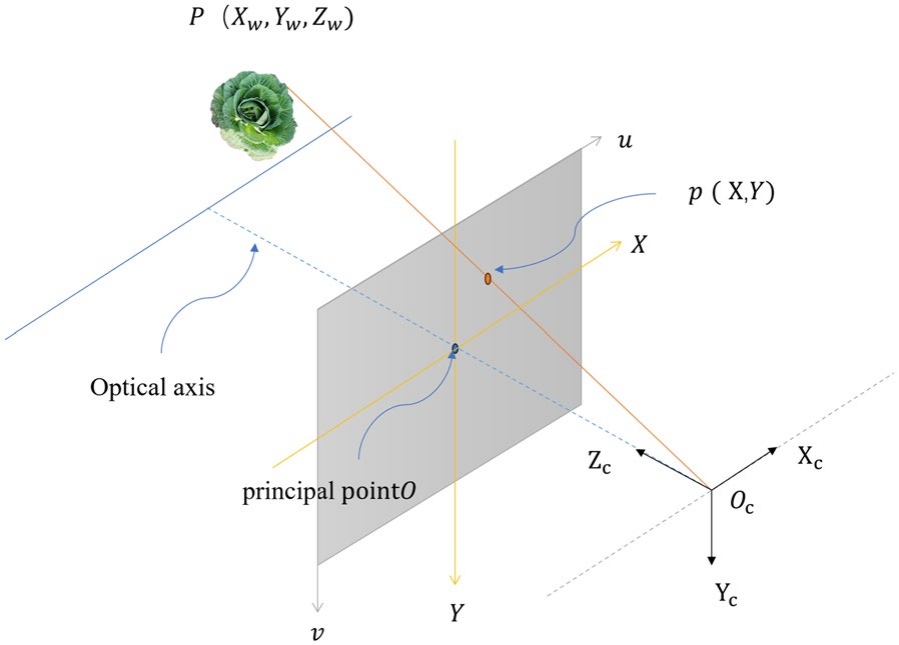
Coordinate diagram

To obtain the position of the cabbage in the three-dimensional coordinate system, coordinate system transformation is needed. As shown in Fig. 14, $P$ (${X}_{w},{Y}_{w},{Z}_{w}$) represents the coordinates of the cabbage in the world coordinate system ${O}_{w}$-${X}_{w},{Y}_{w},{Z}_{w}$; ${O}_{c}$-${X}_{c},{Y}_{c},{Z}_{c}$ represents the camera coordinate system with the optical centre as the origin; $O$-$xy$ is the image coordinate system, with the origin at the intersection of the optical axis and the projection plane, known as the principal point; and uv represents the pixel coordinate system, which is in the same plane as the image coordinate system, with the origin at the top left corner of the projection plane. These four coordinate systems can be transformed using the camera’s intrinsic matrix and extrinsic matrix.

#### Depth image alignment

The D435i image is simultaneously received by a pair of stereo infrared sensors and a colour camera. Due to the position deviation of the two cameras, their corresponding pixels have different positions and cannot be used directly. To use colour image data in target detection, the depth map is aligned to the colour map by coordinate conversion with the following conversion formula:

6 $${T}_{d2c}=\left[\begin{array}{cc}{R}_{w2c}{R}_{w2d}^{-1}& {t}_{w2c}-{R}_{w2c}{R}_{w2d}^{-1}{t}_{w2d}\\ 0& 1\end{array}\right]$$

where ${R}_{w2c}$ represents the rotation matrix of the world coordinate system to the colour coordinate system, ${t}_{w2c}$ represents the offset matrix of the world coordinate system to the colour coordinate system, ${R}_{w2d}$ represents the rotation matrix of the conversion from the world coordinate system to the depth coordinate system of the infrared camera, and ${t}_{w2d}$ represents the offset matrix of the world coordinate system to the depth camera coordinate system.

#### Analysis of the positioning results based on the improved algorithms for cabbage detection

The positioning method mainly achieves the following functions: initializing the depth camera and colour camera; calling the improved YOLOv8n algorithm to detect cabbages in the RGB images; using the pixel position of the detection box as the return value to access the depth image; and converting pixel coordinates into world coordinates and outputting the true three-dimensional coordinates of the cabbages relative to the camera position in the world coordinate system.

**Fig. 15:**
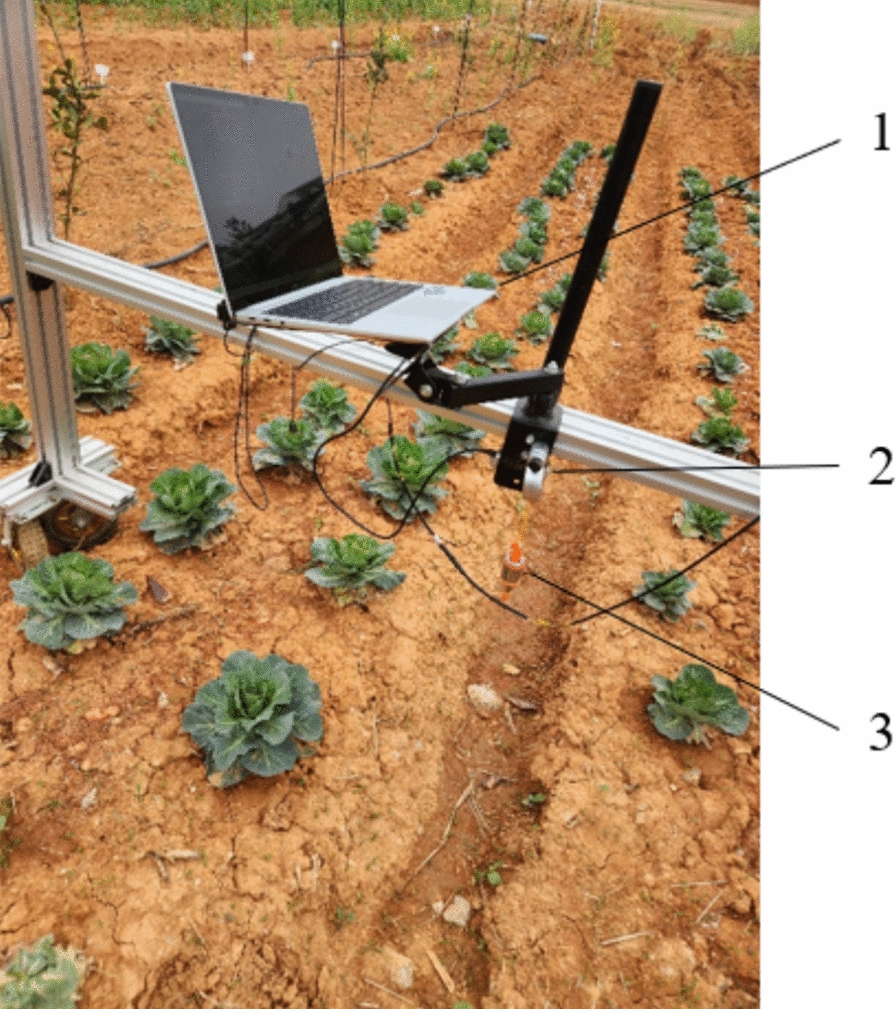
Physical diagram of the test bench

The experimental apparatus included a D435i depth camera, plumb bob, portable computer, measuring tape, etc., for conducting three-dimensional coordinate accuracy measurement experiments in the field. The D435i depth camera is fixed on a test stand with a height of 72.2 cm, and the camera is pointed downwards horizontally. Using this positioning method, three-dimensional measurements of cabbages were obtained. The plumb bob is used to mark the projection of the depth camera on the ground and measure the vertical distance of the depth camera, and the measuring tape is used to measure the coordinates of the projection marked by the plumb bob on the horizontal plane of the cabbage, considering it the actual position. The measured positions are compared with the actual positions to calculate the error. The test stand is moved to measure 20 cabbage plants. The physical image of the test stand is shown in Fig. 15, and the measurement results are shown in Table 4.

**Table 4:** Three-dimensional coordinate measurements of cabbage in the field

ID	Cabbage field location coordinates		
	Real coordinate (mm)	Detection coordinate (mm)	Coordinate error (mm)
1	(− 34, 162, 644)	(− 11, 157, 623)	(23, 5, 21)
2	(20, 61, 560)	(33, 84, 580)	(13, 23, 20)
3	(42, − 36, 537)	(27, − 49, 504)	(15, 13, 33)
4	(18, − 6, 619)	(20, − 20, 630)	(2, 14, 11)
5	(49, 168, 507)	(53, 187, 521)	(04, 19, 14)
6	(2, 135, 554)	(− 12, 132, 565)	(14, 03, 11)
7	(40, 62, 549)	(42, 78, 512)	(2, 16, 37)
8	(21, 44, 622)	(24, 29, 589)	(3, 25, 33)
9	(-12, 15, 613)	(− 6, 13, 577)	(6, 2, 36)
10	(28, 150, 503)	(21, 162, 529)	(7, 12, 26)
11	(53, 60, 592)	(37, 65, 566)	(16, 5, 26)
12	(22, 70, 546)	(40, 77, 542)	(18, 7, 4)
13	(42, 71, 523)	(62, 49, 564)	(20, 22, 41)
14	(11, 59, 520)	(21, 58, 506)	(10, 1, 14)
15	(55, 23, 531)	(49, 13, 498)	(6, 10, 33)
16	(− 1, 54, 554)	(− 24, 76, 515)	(23, 22, 39)
17	(28, 30, 530)	(16, 35, 53.3)	(12, 5, 3)
18	(19, 154, 619)	(40, 149, 568)	(21, 5, 51)
19	(16, 58, 639)	(20, 47, 621)	(4, 11, 18)
20	(41, 140, 588)	(36, 126, 553)	(5, 14, 35)

According to Table 4, the maximum error on the X-axis is 23 mm, with an average error of 11.2 mm; the maximum error on the Y-axis is 25 mm, with an average error of 10.225 mm; and the maximum error on the Z-axis is 51 mm, with an average error of 25.3 mm. These errors are within the acceptable range; thus, the usage requirements have been met. In order to evaluate the degree of data dispersion, standard deviation is introduced to further statistical analysis of the data, the formula for standard deviation is as follows:

7 $$\sigma =\sqrt{\frac{1}{N}\sum_{i=1}^{N} {\left({x}_{i}-\mu \right)}^{2}}$$

where $\sigma$ represents the standard deviation, $N$ stands for the number of constants, $x$ stands for random variable, and $\mu$ represents the average of the variables. The standard deviations of the values on the X-axis, Y-axis and z-axis are 7.7 mm, 7.4 mm and 12.9 mm respectively. This means that the deviation of the measured data on the x and y axes is relatively stable, while the stability of the data on the Z axis is relatively poor.

## Conclusion

In this paper, a field cabbage recognition and positioning method is proposed based on improved YOLOv8n for the detection of cabbage at different growth stages by training models with data from different growth periods. Field cabbage recognition experiments were conducted, and a stand was used to verify precise and accurate positioning, leading to the following conclusions:

1. In response to the morphological changes that occur throughout the entire growth process of cabbage in the field, a detection model specifically tailored for cabbage crops, YOLOv8-cabbage, is proposed. This model employs data augmentation techniques for more comprehensive training, introduces a spatial-based attention mechanism, replaces the C2f layer in the YOLOv8 backbone with a Swin transformer module, and incorporates large convolutional kernels into the backbone network to improve the performance of small object detection and reduce the risk of overfitting. The experimental results demonstrate that the accuracy of the improved algorithm model reaches 95.5%, with an AP of 93.9%. Compared to the original YOLOv8n model, this model gives increases in the accuracy and AP of 3.6% and 5.1%, respectively, indicating significant advantages in accuracy over existing models.
2. After camera calibration, coordinate system transformation, and alignment with the depth map, precise positioning of the cabbage was achieved in both pixel coordinates and world coordinates. The accuracy of the proposed method for three-dimensional cabbage coordinate positioning under field conditions was tested. The average errors in cabbage detection and positioning in the field were (11.2 mm, 10.225 mm, 25.3 mm). Combining the recognition and positioning system of the YOLOv8-cabbage model improved the accuracy of cabbage recognition. The results indicate that the positioning accuracy meets the requirements, providing a reference for cabbage-targeted spraying research.

## Data Availability

The underlying data of this paper cannot be publicly shared as the data is required for further research. These data will be shared with the corresponding author upon reasonable request.
